# β-Lactam/β-Lactamase Inhibitor Combination Antibiotics Under Development

**DOI:** 10.3390/pathogens14020168

**Published:** 2025-02-08

**Authors:** Angeliki Katsarou, Panagiotis Stathopoulos, Iva D. Tzvetanova, Christina-Maria Asimotou, Matthew E. Falagas

**Affiliations:** 1 Alfa Institute of Biomedical Sciences (AIBS), 9 Neapoleos Street, 151 23 Athens, Greece; a.katsarou@aibs.gr (A.K.); p.stathopoulos@aibs.gr (P.S.); c.asimotou@aibs.gr (C.-M.A.); 2 Department of Medicine, Hygeia Hospital, 4 Erythrou Stavrou Street, 151 23 Athens, Greece; 3 School of Medicine, European University Cyprus, 6 Diogenous Street, 2404 Nicosia, Cyprus; i.tzvetanova@euc.ac.cy; 4 Department of Medicine, Tufts University School of Medicine, 145 Harrison Ave, Boston, MA 02111, USA

**Keywords:** antimicrobial resistance, metallo-β-lactamases, β-lactams/β-lactamase inhibitors, cefepime/zidebactam, cefepime/taniborbactam, imipenem/cilastatin/funobactam, meropenem/nacubactam, xeruborbactam/β-lactams

## Abstract

Antimicrobial resistance remains a public health problem of global concern with a great health and financial burden. Its recognition as a threat by political leadership has boosted the research and development of new antibiotics and particularly novel combinations of β-lactams/β-lactamase inhibitors against multidrug-resistant (MDR) Gram-negative pathogens, which remain the major concern in clinical practice. The incorporation of ceftolozane/tazobactam, ceftazidime/avibactam, meropenem/vaborbactam, and imipenem/cilastatin/relebactam has provided new therapeutic options in the treatment of patients with infections due to MDR pathogens. Cefiderocol along with cefepime/enmetazobactam, avibactam/aztreonam, and sulbactam/durlobactam have been recently added to these agents as therapeutic choices, particularly for metallo-β-lactamase producing Gram-negative bacteria. Currently, many combinations are being studied for their in vitro activity against both serine- and metallo-β-lactamases. However, only a few have advanced through phase 1, 2, and 3 clinical trials. Among them, in this article, we focus on the most promising combinations of cefepime/zidebactam, cefepime/taniborbactam, and imipenem/cilastatin/funobactam, which are currently under investigation in phase 3 trials.

## 1. Introduction

Antimicrobial resistance (AMR) has been recognized as a global public health problem by the World Health Organization (WHO) [1]. A recent meta-analysis showed that AMR was associated with almost 5 million deaths in 2019; among these, 1.27 million were directly attributed to bacterial resistance [2]. Particularly, multidrug resistance (MDR) in Gram-negative bacteria, mostly mediated by β-lactamases, is a major problem in clinical practice. The difficulty in combating MDR Gram-negative bacteria is largely attributed to their distinct cell envelope structure compared to Gram-positive bacteria, which impedes antibiotic penetration. In particular, Gram-positive pathogens lack the outer membrane found in Gram-negative bacteria, which contains lipopolysaccharides and functions as a barrier to antibiotic penetration [3].

The impact of AMR is not limited to human health [4]. Along with morbidity and mortality, AMR also brings a great economic burden; according to data from a recent meta-analysis in middle and high-income countries, the healthcare cost associated with drug-resistant infections can vary from USD 2371 to USD 29,289 [4]. In a study in the United States of America regarding healthcare costs associated with MDR bacterial infections in hospitalized patients, considerably high costs were especially attributable to methicillin-resistant *Staphylococcus aureus* [USD 30,998 (95% CI USD 25,272–36,724)] and carbapenem-resistant *Acinetobacter baumannii* [$74,306 (95% CI USD 20,377–128,235)] infections [5]. In 2017, the World Bank estimated that by 2050, in case of high AMR, the global gross domestic product (GDP) could be reduced by 3.8% each year and push 28 million people into poverty [6]. Losses resulting from the impact of drug resistance on livestock could cost global GDP up to USD 950 billion, while the spread of resistant pathogens from livestock to humans could cost up to USD 5.2 trillion [6]. The recognition of the problem led the American presidency a decade ago to characterize AMR in general as a “threat to public health and economy” and the fight against it as “a national security priority” [7]; so, new antibiotic development became one of the US government’s goals for AMR management [8].

The progress in research has brought to the fore of clinical practice new antibiotics, including β-lactam/β-lactamase inhibitor (BL/BLI) combinations, specifically ceftolozane/tazobactam, ceftazidime/avibactam, meropenem/vaborbactam, and imipenem/cilastatin/relebactam [7]. Lately, cefepime/enmetazobactam [9], sulbactam/durlobactam [10], and aztreonam/avibactam are among the recently-approved BL/BLI combinations for use in clinical practice. Drug development has not remained in the realm of BL/BLI combinations, and novel cephalosporins have been incorporated into the pharmaceutical arsenal following the US Food and Drug Administration (FDA) approval of cefiderocol in 2019 [7] and of ceftobiprole medocaril sodium earlier this year [11]. Despite the progress and innovation, MDR Gram-negative pathogens remain a significant public health concern and are listed on the WHO Bacterial Priority Pathogens List for 2024 [12].

β-lactam antibiotics are the largest class of antibiotics; this class is further subdivided into penicillins, cephalosporins, carbapenems, and monobactams. They bind to and inactivate the transpeptidase domain of penicillin-binding proteins (PBPs) and thus inhibit bacterial cell wall synthesis [13]. The most common mechanism of resistance of Gram-negative bacteria to β-lactams is through the expression of β-lactamases, which hydrolyze the amide bond within the β-lactam ring, leading to antibiotic inactivation. β-lactamases are structurally subdivided into four Ambler classes (Class A, B, C, and D) [13]. Functionally, Classes A, C, and D β-lactamases hydrolyze BLs via nucleophilic attack through a conserved serine residue and are thus termed serine-β-lactamases. On the other hand, Class B β-lactamases are known as metallo-β-lactamases (MBLs), since they require Zn^2+^ for BL hydrolysis [13]. The most difficult-to-treat Gram-negative pathogens, e.g., *Pseudomonas aeruginosa* [14] and *Acinetobacter* spp. [15], express extended-spectrum β-lactamases (ESBL), e.g., AmpC-producing Enterobacteriaceae, and/or *Klebsiella pneumoniae* carbapenemase (KPC) or OXA-like carbapenemases, e.g., carbapenem-resistant Enterobacteriaceae (CRE).

In this article, we aimed to focus on BL/BLI combination antibiotics under development in phase 1, 2, and 3 clinical trials, investigating the safety, efficacy, and performance of new drugs compared to the standard of care, respectively. However, at the time of the writing of this article (11/2024), there were no BL/BLI combination antibiotics at the stage of development of phase 2 clinical trials (with published results); thus, we included relevant agents in phase 1 and 3 clinical trials.

To provide a comprehensive overview, we compiled the information into three tables. Table 1 presents BL/BLI combination antibiotics currently under investigation in phase 1 trials, while Table 2 includes those in phase 3 trials. Table 3 presents a detailed insight into the antibiotic class, mechanism of action and antimicrobial spectrum of these agents.

## 2. β-Lactam/β-Lactamase Inhibitor Combination Antibiotics in Phase 3 Trials

### 2.1. Cefepime/Zidebactam

Cefepime/zidebactam is one of the combinations of BL/BLI under development. Cefepime, a fourth-generation cephalosporin [16], has a broad-spectrum activity against Gram-positive and Gram-negative bacteria; it is used for complicated urinary tract infections (cUTIs), intra-abdominal infections, respiratory tract infections, and neutropenic fever [17]. Cefepime alone retains activity against AmpC-producing Gram-negative pathogens [18]. Zidebactam belongs to a new β-lactamase inhibitor category (along with avibactam and relebactam) known as diazabicyclooctanes (DBOs) [19]. In particular, zidebactam is an “enhancer” that binds with high affinity to penicillin-binding protein 2 (PBP2) and inhibits β-lactamases, thereby preventing hydrolysis of cefepime and enhancing its antimicrobial activity [16,20]. The combination of these two agents has proven in vitro activity against Enterobacteriaceae and *Pseudomonas aeruginosa* that produce β-lactamases, including ESBL, KPC, and MBL [21]. However, the in vitro antimicrobial activity of cefepime/zidebactam against *Acinetobacter baumannii* [22], *Stenotrophomonas maltophilia*, *Proteus* species, and *Serratia* seems limited [19]. Interestingly, zidebactam improved cefepime pharmacodynamics [23] in vivo, and the combination effectively reduced carbapenem-resistant *Acinetobacter baumannii* burden in the neutropenic murine lung [24] and thigh [25] infection models.

Cefepime/zidebactam has been reported to be effective in treating patients with extensively drug-resistant *Pseudomonas aeruginosa* infections under compassionate use as salvage treatment [26,27]. More specifically, its use concerned the case of a young adult suffering from acute T-cell leukemia and disseminated infection from extensively drug-resistant (XDR) *Pseudomonas aeruginosa* producing New Delhi metallo-β-lactamase (NDM) [26]. The isolate was resistant to the combinations of ceftolozane/tazobactam, ceftazidime/avibactam, and carbapenems yet susceptible to colistin (polymyxin E). The patient was treated with a combination of polymyxin B and meropenem [26]. However, clinical deterioration with necrotizing ecthyma gangrenosum and lung involvement, along with the polymyxin B-induced neurotoxicity, led to the use of cefepime/zidebactam as a last-resort treatment; the prolonged antibiotic administration along with surgical source control resulted in gradual clinical improvement [26]. Another female patient with a history of bariatric surgery suffering from multi-organ dysfunction after intra-abdominal infection with XDR *Pseudomonas aeruginosa* expressing NDM was successfully treated with the new combination after polymyxin failure [27].

Currently, the combination is being investigated in a phase 3, randomized, double-blind clinical trial (NCT04979806) that is expected to be completed by the end of 2024. It is a multicenter, non-inferiority trial comparing cefepime/zidebactam (at a dose of 2 g of cefepime and 1 g of zidebactam every 8 h) against meropenem (1 g every 8 h) in patients hospitalized for cUTI or acute pyelonephritis. However, pharmacokinetic data in healthy adults showed that plasma and lung concentrations of this drug combination could also support its use for nosocomial pneumonia by susceptible pathogens [28]. Additionally, its use was reported to be safe in patients with renal impairment as long as there is a dose adjustment [16]. Particularly, only local adverse events with erythema and swelling in the injection area have been related to the drug in a single-center study [16]; also, likely associated adverse events in healthy subjects were rash and pruritus [16].

### 2.2. Cefepime/Taniborbactam

Taniborbactam is a boronic-acid-containing β-lactamase inhibitor of β-lactamases of Class A, C, and D, as well as some of Class B (including VIM, NDM, SPM-1, and GIM-1 but not IMP) [29]. The combination of the fourth-generation cephalosporin with the inhibitor provides an extended in vitro activity against CRE and *Pseudomonas aeruginosa* (CRPA), either isolates producing carbapenemase or non-producing, as well as against isolates with resistance to the novel combinations (ceftolozane/tazobactam, meropenem/vaborbactam, ceftazidime/avibactam) [30]. It also exhibited activity against *Pseudomonas aeruginosa* resistant to meropenem, ceftazidime/avibactam, ceftolozane/tazobactam, and meropenem/vabobactam, as well as MDR and difficult-to-treat resistant (DTR) isolates. DTR refers to isolates resistant to fluoroquinolones and β-lactams, excluding the newer BL/BLI ceftazidime/avibactam, ceftolozane/tazobactam, and meropenem/vabobactam [31].

The combination has exhibited in vivo activity against Enterobacteriaceae, *Pseudomonas aeruginosa*, and *S. maltophilia* in murine models of cUTI [32]. A relevant study also demonstrated in vivo activity against Enterobacteriaceae and *Pseudomonas aeruginosa* that were not susceptible to cephalosporin alone in the pneumonia murine model [33]. Cefepime/taniborbactam is the most studied among the novel BL/BLI combinations under investigation. Thus, the positive results from a phase 3 trial led a pharmaceutical company to apply for drug approval. However, the FDA rejected the company’s application in February 2024 [34]. The results from this randomized, non-inferiority trial comparing the combination at a dose of 2 g of cefepime and 500 mg of taniborbactam intravenously vs. 1 g of meropenem every 8 h for the treatment of cUTI were recently published [35]. Cefepime/taniborbactam was proven to be superior to meropenem in terms of microbiologic and clinical success in patients with Gram-negative pathogens susceptible to both agents of the study (70.6% in the cefepime/taniborbactam group vs. 58% of meropenem-treated, 95% CI, 3.1–22.2; *p* = 0.009) [35]. Adverse events were reported at a similar frequency in the combination- and the meropenem-treated patients (35.5% vs. 29%) [35]. In the cefepime/taniborbactam group, the most frequently reported adverse events were headaches, gastrointestinal disturbances, and increased blood pressure [35]. In addition, three patients in this group (*n* = 440 patients received the combination) suffered from *clostridium difficile* infection vs. no cases in the meropenem-treated group (*n* = 217 patients received meropenem) [35].

### 2.3. Imipenem/Cilastatin/Funobactam

Funobactam is a serine-β-lactamase inhibitor (known as XNW4107 in the past) with a spectrum against β-lactamases of Class A, C, and D [36]. Its co-administration with imipenem broadens activity against *Acinetobacter baumannii* and *Klebsiella pneumoniae* resistant to carbapenems; funobactam enhances the activity of imipenem against the above bacteria (previously resistant to imipenem) in vitro and in vivo in mouse models [37]. Currently, two randomized, phase 3 trials are in progress. The first investigates the role of the intravenous combination of imipenem/cilastatin/funobactam at a dose of 500 mg/500 mg/250 mg, respectively, every 6 h vs. meropenem at a dose of 1 g every 8 h for cUTI in hospitalized adults (NCT05204368) [38]. The second trial evaluates the efficacy of intravenous imipenem/cilastatin/funobactam at a dose of 500 mg/500 mg/250 mg, respectively, every 6 h against imipenem/cilastatin/relebactam for the treatment of hospital-acquired pneumonia, including ventilator-associated pneumonia (NCT05204563) [39].

## 3. B-Lactams/B-Lactamase Inhibitors in Phase 1 Trials

### 3.1. Meropenem/Nacubactam

Nacubactam is the fourth agent of the bridged DBO β-lactamase inhibitors [40]. When used alone, it has proven effectiveness against Gram-negative bacteria; its activity may be broadened against Enterobacteriaceae-producing ESBL, KPC, MBL, AmpC, and OXA-48 when the inhibitor is combined with β-lactams [40,41,42]. Consequently, this antimicrobial activity is attributed both to the direct impact on pathogens by targeting PBP2 and, concurrently, by enhancing the action of the second β-lactam agent on PBP3 [41]. Additional activity of the combination against *Pseudomonas* yet not *Acinetobacter* is supported according to a multicenter study trying to determine its in vitro activity against GNB [29].

When the combination was tested in neutropenic mice, its concentration in the human-simulated epithelial lining fluid was effective against Enterobacteriaceae-producing Class A serine carbapenemases; the combination was superior to either agent alone in terms of bacterial density decline [43]. Similarly, the meropenem/nacubactam combination was more efficacious than either agent alone in reducing MDR Enterobacteriaceae isolates in neutropenic mice with cUTI [44], supporting potential clinical use in cUTI.

The pharmacokinetics of the co-administration of the two drugs for up to 2 weeks has also been studied in a non-randomized trial (NCT03174795) in patients with cUTI [45]; however, the results have not been publicly announced. In 2020, the results from a phase 1 clinical trial showed that nacubactam alone or in combination with meropenem was well tolerated in healthy participants; adverse reactions were mostly apparent after the intravenous administration of nacubactam [46]. Among the patients receiving the co-administration of meropenem/nacubactam (at a dose of 2 g/2 g, respectively, every 8 h, intravenously), more than 80% had adverse events similar to the meropenem safety profile; in particular, the most frequently reported were phlebitis, extravasation, headache, and nausea [46].

### 3.2. Xeruborbactam/β-Lactams

Xeruborbactam (previously known as QPX7728) is a cyclic boronate inhibiting both serine-β-lactamases and MBLs [47]. This BLI alone has a broader activity spectrum compared to the second dual inhibitor—taniborbactam—since it is effective against MBL, including IMP [48]. In vitro, xeruborbactam has been proven to be effective against MBL-producing Enterobacteriaceae [47]; when combined with meropenem, it was more potent than cefepime/taniborbactam against MBL-negative CRE (MIC_90_ = 1 μg/mL vs. MIC_90_ = 16 μg/mL) [47]. The drug safety in combination with meropenem has been tested in a phase 1 trial; the results were announced in late 2022 [49]. The trial showed the drug safety and tolerance in healthy adults when administered either alone or in combination with meropenem (the combination was administered at a maintenance dose of 2 g of meropenem/250 mg of xeruborbactam every 8 h intravenously, after a loading dose of 4 g for meropenem and 500 mg for xeruborbactam); the authors pointed out that its favorable pharmacokinetics may also provide the potential for co-administration with other β-lactams [49].

To date, no other clinical trials have been published regarding this combination. Currently, two phase 1 trials are testing the pharmacokinetics of xeruborbactam in combination with other β-lactams, as shown in Table 1. The first is a double-blind randomized controlled trial concerning its administration with cefiderocol (NCT06547554) [50]. The second concerns the administration of xeruborbactam oral prodrug combined with ceftibuten (NCT06079775) [51]. Finally, another pharmacokinetics trial concerning xeruborbactam oral prodrug with ceftibuten in patients with varying degrees of kidney impairment is expected to start in the coming months (NCT06157242) [52].

### 3.3. Meropenem/Pralurbactam

Pralurbactam (also known as FL058) is another novel DBO active against β-lactamases of Class A, C, and D [53]. The first trial testing the safety of the combination of meropenem/pralurbactam in healthy subjects was recently published, showing safety and tolerability [53]. The most common adverse events are gastrointestinal, such as nausea and vomiting [53]. A phase 3, randomized, double-blind, multicenter, positive control trial aimed at comparing the efficacy, safety, and pharmacokinetics of meropenem/pralurbactam (at a total dose of 3 g every 8 h intravenously) to that of ceftazidime/avibactam/metronidazole in the treatment of adult complicated intra-abdominal infections is reported to start this year (NCT06633718).

Other BL/BLI being investigated in phase 1 trials, either completed, with the results pending public announcement, or in progress, are depicted in Table 1.

## 4. Discussion

Antimicrobial resistance remains a global public health problem, as the WHO has pointed out [1], with a great impact in terms of health and financial burden. In clinical practice, the combinations of ceftolozane/tazobactam, ceftazidime/avibactam, meropenem/vaborbactam, and imipenem/cilastatin/relebactam have provided and expanded therapeutic choices against most of the classes of β-lactamases. Notably, most of these BL/BLIs developed during the last decade provide therapeutic options against Class A, C, and D β-lactamases [54]. However, the MBL-producing pathogens (Class B β-lactamases) are a significant problem, since pharmaceutical choices are extremely limited in clinical practice in most countries, making the development of effective antibiotics still an unmet need. Currently, according to Infectious Diseases Society of America 2024 Guidance, the only options in our pharmaceutical armamentarium for MBL-producing Enterobacteriaceae are the combination of ceftazidime/avibactam with aztreonam and the newer cefiderocol [55]. Meanwhile, the novel combination of avibactam/aztreonam has been recently approved by the European Medicines Agency for hospital-acquired pneumonia, complicated urinary tract infections, and intra-abdominal infections by Gram-negative pathogens [56], covering, additionally, MBL-producing bacteria [57]. Combinations under development, such as cefepime/zidebactam, meropenem/nacubactam, and cefepime/taniborbactam, may also exhibit potential activity against Gram-negative bacteria producing β-lactamases, including MBL [29,58,59]. Among these, cefepime/taniborbactam has moved closer to approval after the positive results of a phase 3 trial recently published for the treatment of cUTI.

β-lactamases are enzymes that are further classified into Classes A, B, and D, which are serine lactamases, and Class B, which are metallo-β-lactamases [60]. The zinc ion is a prerequisite for MBL activity, since it opens the β-lactam ring by activating a water molecule [61], inducing, particularly, a carbapenemase function [62]. The rapid appearance of new variants, the gene transferability of encoding genes, the different structure from serine-β-lactamases, and the lack of effective drugs in the market [62] contribute to the difficulty in managing infections by MBLs. Even if BL/BLI is still the leading antibiotic class targeting the pathogens that have been prioritized by the WHO [12], the development of new agents with potential activity against MBL remains of particular interest. Thus, the evolution of drug technology has led us to the promising incorporation of the third generation of β-lactamase inhibitors, known as boronate compounds, such as taniborbactam and xeruborbactam, that could provide beneficial activity against MBL [62]. These agents were added to the already-known third-generation inhibitor, vaborbactam, which in combination with meropenem has been useful for bacteria producing serine-β-lactamases [62,63]. Interestingly, considering the crucial role of zinc ion in MBL activity, chelating agents such as Aspergillomarasmine A may play a role in MBL management [64]. However, the research should be intensified to delve into the exact underlying mechanism of action of the enzymes responsible for antibiotic resistance. In addition, among the questions to be answered are the variable effectiveness of BL/BLI (such as boronates) against the different MBLs, to provide crucial information for further innovation.

Finally, another concern arising from the high cost of new antibiotics is possible inequities in the availability of drugs between low-, middle-, and high-income countries [65]. Expensive novel combinations could make their administration unfeasible in different settings (in terms of socio-economic background) [65]. Furthermore, the AMR burdens vary across geographical regions, even on the same continent (e.g., European region) [66]; thus, these discrepancies in patient management could further increase. Subsequently, the development of novel pharmaceutical agents does not guarantee the treatment of infections by MDR pathogens and the elimination of AMR. The in-depth knowledge of the spectrum and mechanism of action along with antibiotic stewardship targeting the rational use of antibiotics can be the leading edge in the battle against multidrug-resistant pathogens, especially in healthcare settings with limited facilities and resources.

## Figures and Tables

**Table 1:** BL/BLI combination antibiotics under investigation in phase 1 trials (ongoing or completed with no published results).

BL/BLI	ClinicalTrials.gov ID	Study Status	Comments
Cefpodoxime proxetil/ETX0282	NCT03491748	Completed	Healthy subjects
Ceftibuten/ledaborbactam	NCT06665555	Recruiting	Healthy subjects
Cefepime/nacubactam (OP0595)	NCT05887908	Active, not recruiting	Healthy subjects
Aztreonam/nacubactam (OP0595)	NCT05887908	Active, not recruiting	Healthy subjects
Meropenem/ANT3310	NCT05905913	Completed	Healthy subjects
Meropenem/ANT3310	NCT06527677	Recruiting	Subjects with renal impairment
Meropenem/KSP-1007	NCT05226923	Completed	Healthy subjects
Ceftibuten/VNRX-7145	NCT04877379	Completed	Healthy subjects and subjects with renal impairment
	NCT05527834	Completed	Healthy subjects
	NCT05488678	Completed	Subjects with renal impairment
Xeruborbactam/cefiderocol	NCT06547554	Recruiting	Healthy subjects
Xeruborbactam/ceftibuten	NCT06157242	Not yet recruiting	Subjects with renal impairment
	NCT06079775	Recruiting	Healthy subjects

**Table 2:** BL/BLI combination antibiotics under investigation in phase 3 trials (ongoing or completed with no published results).

BL/BLI	ClinicalTrials.gov ID	Study Status	Comparator	Site of Infection
Cefepime/zidebactam	NCT04979806	Recruiting	Meropenem	cUTI
Cefepime/nacubactam	NCT05905055	Recruiting	Aztreonam/nacubactam	cUTI, AP, HABP, VABP, and cIAI
Aztreonam/nacubactam	NCT05905055	Recruiting	Cefepime/nacubactam	cUTI, AP, HABP, VABP, and cIAI
Imipenem/cilastatin/funobactam	NCT05204368	Not yet recruiting	Meropenem	cUTI
Imipenem/cilastatin/funobactam	NCT05204563	Recruiting	Imipenem/cilastatin/relebactam	HAP, VABP

Abbreviations: AP, acute uncomplicated pyelonephritis; cIAI, complicated intra-abdominal infection; cUTI, complicated urinary tract infection; HABP, hospital-acquired bacterial pneumonia; VABP, ventilator-associated bacterial pneumonia.

**Table 3:** Antibiotic class, mechanism of action, antimicrobial spectrum of BL/BLI under development.

BL/BLI	Antibiotic Class	Mechanism of Action	Antimicrobial Spectrum
Cefepime/taniborbactam	4th-generation Cefalosporin/boronic-acid-containing β-lactamase inhibitor	Inhibits bacterial cell wall synthesis by binding to PBPs/inhibits β-lactamases of Classes A, C, D, and some of Class B (VIM, NDM, SPM-1, GIM-1).	Extended activity in vitro against carbapenem-resistant Enterobacterales (CREs) and *Pseudomonas aeruginosa* (CRPA), including isolates resistant to novel BL/BLI combinations (e.g., ceftazidime/avibactam). Effective against metallo-β-lactamase (MBL)-producing Enterobacterales and MDR/DTR pathogens.
Imipenem/funobactam	Carbapenem β-lactam/serine β-lactamase inhibitor	Inhibits bacterial cell wall synthesis by binding to PBPs/inhibits serine β-lactamases of Classes A, C, and D.	Broadened activity against carbapenem-resistant *Acinetobacter baumannii* and *Klebsiella pneumoniae*. Effective in vitro and in vivo against imipenem-resistant strains.
Cefepime/zidebactam	4th-generation cephalosporin/diazabicyclooctane (DBO) β-lactamase inhibitor	Inhibits bacterial cell wall synthesis by binding to PBPs/binds to PBP2 and inhibits β-lactamases.	Effective in vitro against Enterobacterales and *Pseudomonas aeruginosa* producing β-lactamases (e.g., ESBL, KPC, MBL). Limited in vitro activity against *Acinetobacter baumannii*, *Stenotrophomonas maltophilia*, *Proteus*, and *Serratia*.
Meropenem/nacubactam	Carbapenem/bridged diazabicyclooctane (DBO) β-lactamase inhibitor	Inhibits bacterial cell wall synthesis by binding to PBPs/targets PBP2, inhibits β-lactamases, and enhances meropenemactivity on PBP3.	Active against Gram-negative bacteria, including ESBL-, KPC-, MBL-, AmpC-, and OXA-48-producing Enterobacterales. Activity against *Pseudomonas aeruginosa* but limited for *Acinetobacter baumannii*.
Xeruborbactam/β-lactams	Cyclic boronate inhibitor/variable	Inhibits serine- and MBL, including IMP/inhibits bacterial cell wall synthesis.	Effective against MBL- and serine-β-lactamase-producing Enterobacterales. Superior activity against MBL-negative CRE compared to other combinations like cefepime/taniborbactam.
Meropenem/FL058	Carbapenem/diazabicyclooctane (DBO) β-lactamase inhibitor	Inhibits bacterial cell wall synthesis by binding to PBPs/inhibits β-lactamases of Classes A, B, and D.	Active against Gram-negative bacteria, including Class A, B, and D β-lactamase producers.

Abbreviations: BL: β-lactam, BLI: β-lactamase inhibitor, PBPs: penicillin-binding proteins, ESBLs: extended-spectrum β-lactamases, KPC: *Klebsiella pneumoniae* carbapenemase, NDM-1: New Delhi metallo-β-lactamase-1, VIM: Verona integron-encoded metallo-β-lactamase, IMP: imipenemase metallo-β-lactamase, SPM-1: São Paulo metallo-beta-lactamase-1, GIM-1: German imipenemase-1.

## Data Availability

Not applicable.

## Abbreviations

AMR	antimicrobial resistance
BL/BLI	β-lactam/β-lactamase inhibitors
cUTI	complicated urinary tract infections
ESBL	extended-spectrum β-lactamases
GIM-1	German imipenemase-1
IMP	imipenemase metallo-β-lactamase
KPC	*Klebsiella pneumoniae* carbapenemase
MBL	metallo-β-lactamase
MDR	multidrug resistance
NDM	New Delhi metallo-β-lactamase
SPM-1	São Paulo metallo-beta-lactamase-1
VIM	Verona integron-encoded metallo-β-lactamase
WHO	World Health Organization
XDR	extensively drug-resistant
